# Mass Spectral Imaging to Map Plant–Microbe Interactions

**DOI:** 10.3390/microorganisms11082045

**Published:** 2023-08-09

**Authors:** Gabriel D. Parker, Luke Hanley, Xiao-Ying Yu

**Affiliations:** 1 Department of Chemistry, University of Illinois Chicago, Chicago, IL 60607, USA; 2 Materials Science and Technology Division, Oak Ridge National Laboratory, Oak Ridge, TN 37830, USA

**Keywords:** mass spectrometry imaging, plant–microbe interactions, MALDI, SIMS, metabolomics, sample preparation, metabolites, machine learning, multivariant analysis

## Abstract

Plant–microbe interactions are of rising interest in plant sustainability, biomass production, plant biology, and systems biology. These interactions have been a challenge to detect until recent advancements in mass spectrometry imaging. Plants and microbes interact in four main regions within the plant, the rhizosphere, endosphere, phyllosphere, and spermosphere. This mini review covers the challenges within investigations of plant and microbe interactions. We highlight the importance of sample preparation and comparisons among time-of-flight secondary ion mass spectroscopy (ToF-SIMS), matrix-assisted laser desorption/ionization (MALDI), laser desorption ionization (LDI/LDPI), and desorption electrospray ionization (DESI) techniques used for the analysis of these interactions. Using mass spectral imaging (MSI) to study plants and microbes offers advantages in understanding microbe and host interactions at the molecular level with single-cell and community communication information. More research utilizing MSI has emerged in the past several years. We first introduce the principles of major MSI techniques that have been employed in the research of microorganisms. An overview of proper sample preparation methods is offered as a prerequisite for successful MSI analysis. Traditionally, dried or cryogenically prepared, frozen samples have been used; however, they do not provide a true representation of the bacterial biofilms compared to living cell analysis and chemical imaging. New developments such as microfluidic devices that can be used under a vacuum are highly desirable for the application of MSI techniques, such as ToF-SIMS, because they have a subcellular spatial resolution to map and image plant and microbe interactions, including the potential to elucidate metabolic pathways and cell-to-cell interactions. Promising results due to recent MSI advancements in the past five years are selected and highlighted. The latest developments utilizing machine learning are captured as an important outlook for maximal output using MSI to study microorganisms.

## 1. Introduction

A mass spectrometer consists of at least three components: a desorption/ionization source, mass analyzer, and ion detector. The mass analyzer and ion detector in mass spectral imaging (MSI) are under a vacuum; some desorption/ionization sources are a under vacuum, while others operate at atmospheric or intermediate pressures [1]. Ion generation is accomplished when material from the sample surface is volatilized and ionized into the gas phase. This process can be performed in multiple ways. Once the ions are created, they then move through the mass analyzer to the mass detector, where the ion signal is converted into an electrical signal. The mass spectrum is generated by the interpretation of the electrical signal generated [2]. 

Mass spectrometry has advanced significantly within the past decade, and it is one of the most widely used analytical platforms. Among MS techniques, MSI or imaging mass spectrometry has advanced the field further and led to wide applications in geological, biological, and medicinal research [3,4]. Matrix-assisted laser desorption ionization (MALDI) mass spectrometry and secondary ion mass spectrometry (SIMS) are two of the leading MSI techniques used in investigations in biology and microbiology. Other techniques, such as desorption electrospray ionization (DESI), laser ablation electrospray ionization (LAESI), laser desorption postionization (LDPI) [5], and secondary neutral ionization (SNMS), are also used in biology and microbiology research [3,6,7,8,9,10]. 

In MSI, an image is created by collecting spectra from different parts of the sample surface in succession. This can be achieved by moving the target specimen so that the desorption/ionization source may examine or investigate different sections of the sample. Moving of the sample is typically accomplished by mounting the sample on a motorized stage, movement of the sample using a piezo stage, manually, or by translating the desorption/ionization probe (i.e., focused laser or ion beam) while the sample remains in place. Once ions are generated, they are extracted into the mass analyzer and detected using a mass analyzer and ion detector, which can vary with the specific configuration of the instrument. Unlike MS used in the analysis of bulk samples, each mass spectrum collected for individual spots analyzed corresponds to pixels that are used in mass spectral images. A range of pixels can be chosen in MSI data acquisition. The detection area, or image area, is also defined by the user and is set before experiments. Rastering over a large area and having smaller spaces between pixel generation will lead to the development of a spatially resolved image that contains sample specific information [6]. Private and public source software allows users to interact with these data by assigning relative distributions to a map of mass spectra for visualization, containing different intensities of mass-to-charge (*m*/*z*) ratios [6].

**Figure 1:**
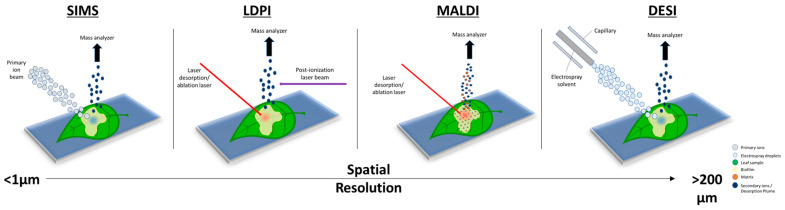
Overview of the different desorption/ionization methods used for mass spectrometry imaging (MSI) to map plant–microbe interactions. The scale bar shows the spatial resolution of each technique, from sub-micrometer (μm) to greater than 200 μm. A higher spatial resolution corresponds to higher-quality mass spectral images.

MSI has become an important tool for biologists and microbiologists alike over the past two decades due to the molecular information collected and contained within the spectral images [11,12]. MSI is powerful because it offers enhanced visualization and spatial resolutions, offering specificity, sensitivity, and temporal information [6,7,13]. Figure 1 shows the main MSI techniques and their corresponding spatial resolutions. Plant–microbe interactions have evaded intact molecular analyses for many years due to the nature and scale in which these interactions occur. MSI, on the other hand, can offer a solution to most, if not all, of the problems encountered with more traditional approaches. A growing concern, as population growth continues to rise, is biomass production and crop sustainability, which is a subject that has a close dependence on plant–microbe interactions. It is estimated that >100 bacterial cells exist per gram of soil [14]. Bacteria form microbiomes for the surrounding plant species, typically colonizing the rhizosphere, rhizoplane, phyllosphere, spermosphere, and endosphere [14,15]. The plant–microbe interactions can either be mutualistic or parasitic depending on the plant–soil feedback [16]. Mapping the biological metabolic pathways and understanding the interactions that microbes have with plants at the molecular level is possible with the use of MSI. The information gained, either quantitative or qualitative, can be far more valuable and relevant compared to standard mass spectrometry measurements of a homogeneous bulk sample [17]. 

MSI has been used to study plant–bacteria interactions and gained more interest in recent years. Specifically, there have been approximately 35 articles covering this topic of plant–microbe interactions since 2004 and six reviews since 2014, according to Web of Science survey results. Confocal laser scanning microscopy (CLSM) is the primary method used for imaging plant–microbe interactions due to its ease of access and reasonable spatial information [18]. Boughton et al.’s review of MSI for plant biology offered a comprehensive overview of MSI capabilities and advances made with the different imaging techniques up to 2015 [3]. Musat et al. tracked microbial interactions with different hosts, plant or animal, via NanoSIMS in a review in 2016 [19]. In 2017, Ho et al. covered the different applications of MSI and detailed microbe–microbe interactions as well as plant–microbe interactions [20]. While MSI has not been a mainstream method for the study of plant–microbe interactions, MSI can provide insights into metabolic pathways, metabolite identification, plant-growth-promoting bacteria, and biotic stress. 

This mini review covers the advances in plant–microbe interactions with a focus on the past three to five years but attempts to cover relevant publications over the last decade. We offer a high-level tutorial of popular MSI techniques and specify the nuances that each instrument has for imaging plant–microbe interactions. This review emphasizes what is available with commercial MSI instrumentation and does not cover fs-LDPI-MS or fs-LDI-MS. We highlight recent advances in sample preparation and imaging applications. The aspect of soil incubation in the microbiome plays a large role in the plant–microbe interaction. Representative results are selected based on a literature survey and presented in this review. Finally, the outlook and recommendations of using MSI in plant–microbe interaction research are put forth, including a brief section on machine learning (ML) in big data analysis. 

## 2. Microbial Biosphere and Instrument Considerations

### 2.1. Microbial Interactions in the Biosphere

**Figure 2:**
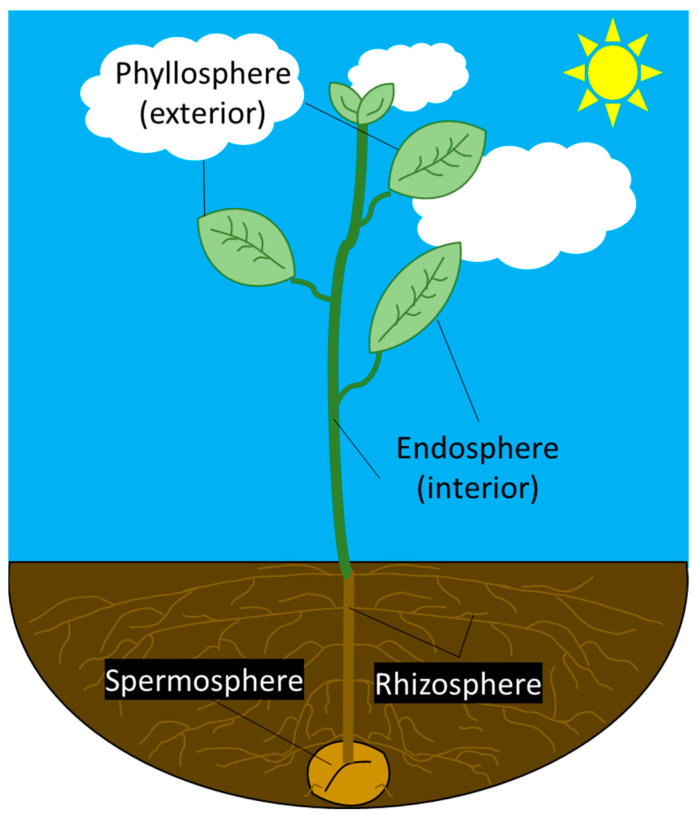
Simplified schematic of a plant system that includes the major spheres for which MSI research has been conducted within the past five years. The region of greatest interest is the rhizosphere, where bacteria and plant root systems interact. Note: The spermosphere here does not accurately represent the progress with which a plant blossoms.

MSI has been used to study plant–microbe interactions in the rhizosphere and endosphere, but few studies are available about the phyllosphere and spermosphere. The latter are also home to microbes and affect plants [15,21,22]. Among the different “spheres,” there are different and diverse microbiota, which affect the plants in various ways. The rhizosphere is the thin layer of soil that surrounds the plant roots, hosting most of the soil microbial growth with complexity under active study [23,24]. Bacilli living within the soil have been shown to protect their host roots from pathogenic microorganisms [23]. The plant root system has been well described in the literature and its importance lies in the efficiency of water and mineral uptake, promoting plant growth and sustaining an environment that allows bacteria to flourish [25]. While the rhizosphere offers bacterial abundance and activity, there is much lower diversity among the bacteria within this zone compared to the bulk soil [26]. Figure 2 shows a simplified version of a plant growing from a seed to a sprouted plant, covering the four main regions in this review. 

The endosphere is the inner tissue region of a plant, such as the inner stem, leaves, and branch system. Bacteria living in the endosphere are referred to as endophytes [27], which have been categorized into two subgroups: ‘obligate’ and ‘facultative’. Obligate endophytes are defined as bacteria that have a dependence on the plant metabolism for survival, being spread by vertical transmission or by the activity of vectors [28]. The facultative endophytes are classified based on whether they have lived outside the host at any time within their lifecycle or are recruited from nearby microbial communities, such as those within the rhizosphere [28]. The endosphere bacteria coevolve with the plant by means of host plant recognition and selection by exudate signaling communication in order to provide homeostatic association [15]. 

The phyllosphere comprises any aerial part of the plant and its respective surfaces. This region of a plant is regarded as a harsh environment for microbes to live in due to inconsistencies with water availability, nutrient abundance, and high UV exposure [29]. Generally, bacteria in this region are associated with nitrogen fixation, the biosynthesis of phytohormones, and protection against pathogenic microbes [15,29]. The predominant area of this region is the leaf surface [30]. The survival of microbial communities in the phyllosphere is dependent on their capability to develop resistance mechanisms, such as UV radiation protection, the production of extracellular polysaccharides, and biosurfactants to promote attachment to surfaces [30]. 

The spermosphere is the seed surface where interactions take place between the microbial communities, soil, and seed germination [31]. The spermosphere area is much smaller compared to the rhizosphere, endosphere, or phyllosphere, but interactions between the spermosphere and the surrounding microbial communities can dramatically impact the growth, maturation, and health of the plant [31].

Microbes can be symbiotic, mutualistic, or parasitic when interacting with the host plant. Symbiotic microbes are also known as plant-growth-promoting microorganisms and have been known to regulate growth factor concentrations, control pathogens, and increase nutrient availability within the host plant [21]. Common rhizobacteria are *Bacillus*, *Pseudomonas*, *Enterobacter*, *Acinetobacter*, *Burkholderia*, *Arthrobacter*, and *Paenibacillus*, all of which are also plant-growth-promoting bacteria [26]. The interactions between the host plant and surrounding microbes are considered some of the most important and complex aspects when studying microbial communities, because evidence indicates that the growth, tolerance, and health of plants are dependent on the microbiome associated with the host plant [21]. Chemical communications between microbes and plants are established by the release of primary and secondary metabolites, which can alter root–root interactions, nutrient availability, microbe accumulation, and biofilm formation [26]. Metabolites are enzymatic catalysts of biological processes and can have a complex and wide distribution throughout a plant [32]. Secondary metabolites produced by microbes interacting with the host plant typically act as nutrient uptake agents [27]. As discussed, there are at least two main challenges in mapping the microbe and plant interactions. First, there are several complex biointerphases and each warrants specific attention. Second, the detection and speciation of metabolites are difficult due to the complexity of microbiomes in nature. Clearly separating coupled events is challenging to fully understand cell-to-cell interactions between microbes and host plants. 

### 2.2. MSI Instrumentation for Imaging Bacterial–Plant Interactions

MSI is a field characterized by rapid adaptation, new technological development, and diverse applications. Some aspects of this group of instruments that continue to improve are their spatial resolution, detection selectivity, and user software for data interpretations [33]. Resolution and mass accuracy are important parameters when considering a technique for analyzing plant–microbe interactions. The lateral or spatial resolution, terms that are used interchangeably, refers to the spacing at which two features of an image are considered separate and distinct [34], and mass accuracy refers to how close the *m*/*z* value peak is to the correct decimal point. The higher the image resolution and mass accuracy, the clearer the information within the mass spectrum image. Achieving both a high image resolution and mass accuracy is often challenging in MSI, yet there have been advances using delayed image extraction that mitigate this difficulty [35].

Table 1 summarizes different mass spectrometry techniques’ lateral resolutions, mass accuracy, and mass resolutions. Among the known MSI techniques, SIMS offers the highest lateral resolution. However, the main challenge in biofilm, plant, and microbiome analysis is that no single technique (e.g., MALDI, DESI, or SIMS) covers the length scale and mass scale simultaneously. Each technique has its own advantages and disadvantages. SIMS can provide the highest lateral resolution with a high mass resolution and high mass accuracy. However, vacuum-based ionization techniques such as SIMS have limitations in terms of the samples that can be introduced and studied due to outgassing materials, substrates, or liquid samples. Furthermore, samples under a vacuum are required to be dried before the analysis takes place, but drying biofilms, for example, can lead to flaking and chipping from the substrate, as well as loss of the native structure. Atmospheric pressure techniques (i.e., DESI) overcome these difficulties, but have a significant trade-off in spatial resolution, namely tens of micrometers compared to the sub-micrometer spatial resolution sometimes possible in SIMS. Therefore, the choice of MSI technique when planning an experiment depends on the target information. Knowing the analysis objectives and these MSI key factors will help to determine the most suitable method to employ. 

When designing an experiment, not only is the ionization source important but choosing a mass analyzer is equally important in obtaining the desired mass accuracy and mass resolution of the data. There are essentially five types of mass analyzers that are currently popular: quadrupole, ion trap, Orbitrap, time-of-flight, and Fourier transform ion cyclotron resonance (FT-ICR) [36]. The combination of mass analyzers with additional ion optical components (i.e., quadrupole time-of-flight or QToF) increases the capabilities for mass identification by tandem MS (analyses that identify ions via collision-induced dissociation) [36]. Fourier transform methods, such as Orbitraps or FT-ICR mass analyzers, have phenomenal mass resolving power [36]. Typically, the time-of-flight (ToF) mass analyzer is coupled with either MALDI or SIMS due to the broad *m*/*z* range, high sensitivity, and acceptable mass resolving power (see below). Reflectron and multipass ToF mass analyzers can attain mass resolving power of >50,000 or greater [37]. Commercially available instrumentation such as the MALDI ToF and ToF-SIMS, has high sensitivity, tandem MS, and a high mass resolution, with which home-built instruments cannot compete for the study of chemically complex biological samples. 

**Table 1:** MSI ionization sources, mass analyzers, and parameters used for plant, bacterial, biological, or other related applications within the past decade.

	Analysis Technique	Lateral/Spatial Resolution	Mass Analyzer	Mass Accuracy	Mass Resolution	Home-Built or Commercial	Field of Study	References
SIMS	SIMS, nanoSIMS, liquid SIMS	0.12–0.5 µm	ToF and Orbitrap	<0.2–1 ppm	unit mass 240,000	Commercial, home-built	Plant, biological, bacterial, and plant–microbe interaction research	[35,38,39,40,41,42,43,44,45,46]
LDI/LDPI	fs-LDI, ns-LDPI, fs-LDPI	2–100 µm	ToF	330–340 ppm	500–30,000	Home-built	Geological, biological, and bacterial research	[4,33,47,48,49,50]
MALDI	MALDI, MALDI-FTICR, AP MALDI, MALDI 2	0.6–150 µm	ToF, Orbitrap, FT-ICR, QToF	0.2–2 ppm	9000–160,000	Home-built, commercial	Plant, biological, bacterial, and plant–microbe interaction research	[23,51,52,53,54,55,56]
ESI/DESI	DESI and LAESI	40–200 µm	Orbitrap, Qtrap, and microToF	≥5 ppm	10,000–70,000	Home-built, commercial	Plant, bacterial, and plant–microbe interaction research	[10,57,58]
Other	Liquid microjunction surface sampling probe, VUV gas discharge lamp, laser ablation, and solvent capture by aspiration/ESI, and LAAPPI	70–260 µm	Qtrap, FT-ICR, Q ToF	≥15 ppm	10,000–400,000	Home-built	Plant, biological, bacterial, and plant–microbe interaction research	[24,57,59,60,61]

### 2.3. Data Analysis Considerations for MSI

Data analysis depends on the information that the analyst wishes to receive and uncover. For example, the quantification and verification of analyte identifications can be challenging when only using standard mass spectrometry. Tandem MS can increase the depth of coverage and the sensitivity and verify the identification of a large number of chemical species [3,62].

Sensitivity can be affected by the noise that is associated with the spectra. Noise can be introduced from multiple sources, such as the electrical system, ion detection, and chemical background, as well as the matrix [63]. Adjusting the signal-to-noise ratio is critical for good spectral results. MS generates large datasets and MSI creates even more data. 

There are both public and commercial software programs developed for general MSI data analysis, such as BioMap (Novartis, Basel, Switzerland) [64] and MSiReader (North Carolina State University) [65], or instrument-specific codes (i.e., SurfaceLab for ToF-SIMS, IONTOF, GmbH, Münster, Germany). Custom software coded in python or MATLAB is common [3]. The manual processing of large amounts of data and thousands of spectra is extremely difficult. Multivariant analysis, specifically principal component analysis (PCA), is typically employed for the analysis of SIMS spectra and spectra obtained by other MS imaging methods [33,66]. PCA is a mathematical algorithm that looks at the variation within the MSI data. It decreases the dimensionality inherent within the data themselves by identifying directions or principal components where the variation in the data is highest [67]. After PCA is performed, the data can be plotted and visualized to identify similarities or differences within large datasets [67]. Analysis using PCA has its benefits by identifying areas within the spectra that are affected by matrix effects so that other more important features can be uncovered [68]. PCA is a useful tool for deciphering SIMS imaging and spectral data that pertain to plant–microbe interactions since there are many inherent variables. For example, PCA has been used to study the effects of Gram-positive and Gram-negative plant-growth-promoting bacteria on seedlings [68]. 

## 3. Sample Preparation Techniques for Plants and Microbes

**Figure 3:**
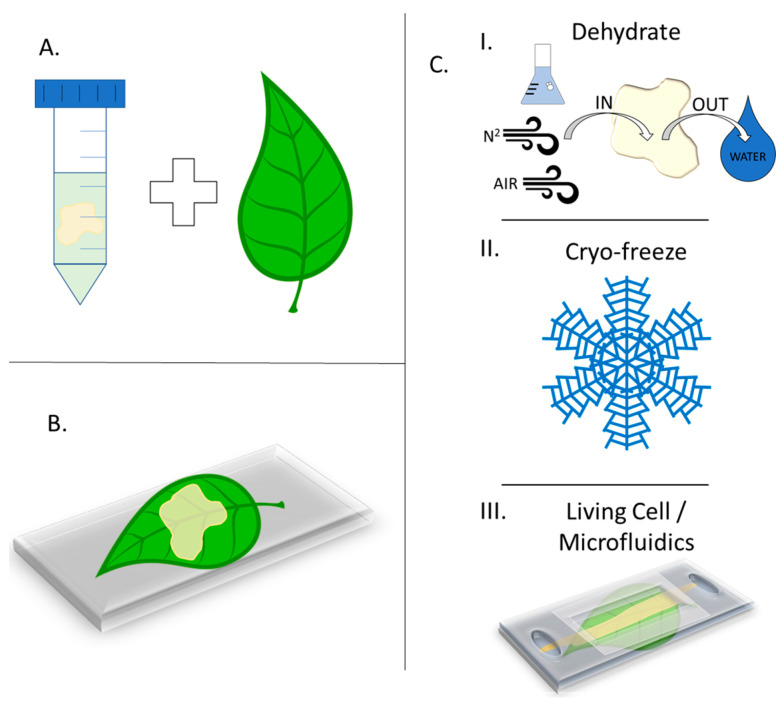
Schematics showing (**A**) culturing the bacterial biofilm on the sample substrate or overlaying it later; (**B**) securing the plant and biomaterial onto an analysis plate or coupon before analysis preparations that include drying, freezing, or the use of microfluidic devices; (**C**) three common methods for plant–microbe sample preparation, including the dehydration of the biofilm on the plant (I), cryo-freezing both the plant and biofilm (II), and native (live) sample preparations for intact analysis (III).

Sample preparation for MSI of in situ analytes and samples could be a limiting factor when deciding on ionization and analysis methods [22]. While information for sample preparation methods regarding plant tissue is known to be limited, the preparation required is a critical step associated with the quality and authenticity of the imaging results [69]. Generally, there are three ways that samples can be prepared, namely drying, freezing, cryo-freezing, or using living samples. Figure 3 gives an overview of these methods and the general process by which a sample is prepared. 

Drying the sample is a common practice for plant or bacterial MSI analysis. The use of a nitrogen stream to dry the sample for a designated period has been applied to prepare *Brachypodium* seeds before having them sectioned into segments for ToF-SIMS analysis [35]. Nonetheless, a necessary step before drying is the desalination of bacteria and biofilms. Desalination is crucial, especially for SIMS, because any salts left over from the media can have a strong matrix effect. In other words, intense signals from the salts could mask signals of biological importance. 

Another method of fixing a sample prior to analysis is embedding it in resin. This sterilizes and dries the sample so that a cross-section can be cut, and analysis can take place at an interface—for example, the plant–microbe biofilm interface. Distortion of ultrafine cellular structures and microbe colonies is avoided by gradual dehydration and infiltration when performing resin embedding [39]. Vacuum drying is another method recently used for SIMS. Liu et al. used the load lock to dry the sample under a vacuum before introducing/loading it into the main analysis chamber [40]. Although convenient, drying in the load lock can have negative effects on the instrument and is not the best practice for sample preparation. Drying inside a laminar flow hood is the preferred option for drying biofilms and plant species [70]. 

Freeze drying and cryo-freezing are other approaches used for sample preparation in SIMS, MALDI, and LDI. Rapid freezing with liquid nitrogen quickly secures a sample that has been used—for example, on *M. polymorpha* plant samples, where the biological samples were allowed to dry on the MALDI sample plate under a laminar flow hood before adding the matrix [51]. Peltier cooling stages can also be used to freeze leaf sections before MSI for analyses performed at atmospheric pressure [10].

The difference between regular drying and freezing/cryo-freezing is whether the water molecules are trapped within the sample to be analyzed. Freezing preserves the water molecules but distorts them as ice crystals form. Drying dehydrates the sample, leading to a distorted biofilm or plant species. Ideally, the optimal way to study how plants and microbes are interacting is by analyzing and imaging them in their native live state. Some techniques, such as DESI and LAESI, can analyze living samples due to their operation in atmospheric pressure. Typically, living biofilms in the liquid state are difficult to study using vacuum techniques; thus, mostly dried or frozen samples have been used for various MSI analyses [46]. 

Yu and coworkers developed a microfluidic device to overcome this challenge [71]. The device, termed SALVI, a system for analysis at the liquid–vacuum interface, is capable of maintaining liquids and living biological specimens with high vapor pressure in high-vacuum instruments, such as a scanning electron microscope or a time-of-flight mass analyzer [71]. The SALVI microfluidic device has been used to study plant–microbe interactions by mass spectrometry imaging [35,42,46,68,72]. While the main peaks between dry samples and viable samples in liquid using SALVI were demonstrated to be similar, there was a reduction in mass accuracy and resolution when using the SALVI, in part because the ToF-SIMS imaging mode was used [46]. When using SALVI in liquid SIMS, the imaging mode with a finely focused capability is necessary. The ToF-SIMS imaging mode has an inherently lower mass resolution and mass accuracy compared to the spectral mode. The latter has much a higher mass resolution and mass accuracy, and it is often used in studying cells and organics in static SIMS applications.

One major issue that arises with sample preparation and analysis is the matrix effect when utilizing MSI. Matrix effects arise differently within each desorption/ionization technique, but often arise interfering with the ionization intensity signal within the mass spectrum. Ion yields for solutes depend on the matrix as well as the identity of the solute and its contribution to the sample surface, ultimately leading to non-linearity with the solute concentration, which results in quantification difficulties [73,74,75,76,77]. Another aspect causing matrix effects is the charge transfer event, which occurs in SIMS, MALDI, and DESI. For example, analyte molecules competing for charge or changes within the matrix caused by cation concentrations have been observed in MSI [76]. Mitigation of the matrix effect is critical for MSI data to obtain accurate spatial distributions of the molecules within the sampled region and to provide accurate mass spectrometry images. In relation to matrix effects, when performing bacterial-based sample analysis, external conditions such as humidity also affect how matrix co-crystallization is produced when using MALDI [78]. 

One aspect that is often overlooked during MSI sample preparation is sample sterilization and disinfection. This is always a necessary step that usually is not mentioned because it is considered to be arbitrary. However, the recent literature has shown that methods for sterilization and disinfection, such as autoclaving, dry heating, ethanol submersion, and other advanced methods including plasma cleaning and UV irradiation, are not fool-proof methods. Sterilizing the sample holder, sample plate, and the sample itself is often necessary to prevent contamination from other strains of bacteria. Kummer et al. showed, for two strains of *Staphylococcus*, that UV treatment and ethanol sterilization left behind ~1 × 10^5^ CFUs/mL of live cells, while an autoclave left behind ~2 × 10^5^ CFUs/mL after heat treatment for 2 h at 500 °C [79]. The live vs. dead bacteria were imaged by introducing fluorescent staining and showed that UV and ethanol, after heat treatment, were more effective methods for sterilization than autoclaving for these *Staphylococcus* strains. Another study by Kasmaei et al. tested several sterilization methods on grass samples with epiphytic microflora: ethanol, cold shock, ethanol/sodium hypochlorite, a neutral detergent, and both moist and dry heat [80]. Their results suggested that dry heating samples at 121 °C for 20 min was the best method of sterilization, while ethanol and the neutral detergent were only effective against lactic acid bacteria. The cold shock was ineffective and treatments using ethanol and NaClO were inconclusive. A study conducted by Chansoria et al. found that the autoclaving of samples was effective for sterilizing biomaterials, alginate in this study, irrespective of the bacterial type, whereas ethanol and UV sterilizations were dependent on the bacteria type and load [81]. Sterilization is an important aspect for reliable sample preparation and standard methods should not be all lumped together as 100% effective. Understanding the biochemical characteristics of the strains of bacteria used during studies is necessary in choosing the appropriate method to apply for the sterilization of samples such that contamination is not an issue. Moreover, the use of more than one method of sterilization and disinfection is encouraged. 

The preparation of samples should be considered well before an experiment as it can affect which method of ionization can be used for the analysis. The sample needs to be conductive otherwise there will be a build up of charge leading to sample charging and poor ionization. The way that the preparation is handled will also affect the outcome of the experiment, and it is imperative to carefully consider how drying, freezing, or imaging under live conditions will achieve the desired analysis goals.

## 4. MSI Ionization Techniques to Investigate Plant–Microbe Interactions

### 4.1. Secondary Ion Mass Spectrometry

Secondary ion mass spectrometry (SIMS) is a method that uses a primary ion beam to desorb analytes from the sample surface, in turn creating a plume of secondary ions and neutrals. While there are many configurations of SIMS instruments, the fundamental basis remains the same: the measurement of the mass and intensity of secondary ions produced while under a vacuum after sputtering the sample surface with an ion beam or neutral beam [82]. Early experiments with ion desorption can be traced back to R. F. K. Herzog and F. P. Viehbock, where they experimented with new ion sources and the sputtering of a sample. Then, in 1957, D.G. Bills demonstrated the desorption of ions from metal surfaces after the bombardment of the metal surface with nitrogen ions or low-energy electrons, stemming from earlier works by Herzog [83]. In 1963, the sputtering ion source using concentrated argon ions to bombard the sample surface for secondary ion desorption was introduced by Liebl and Herzog [84]. However, it was not until 1972, when Huber, Selhofer, and Benninghoven demonstrated that the SIMS technique under an ultra-high vacuum (UHV) was able to measure both positive and negative ions, and to acquire profiles of multilayer films, that the basis for the modern SIMS instruments was set [85]. SIMS imaging was not far behind after sputtering sources were announced, and early SIMS imaging could be attributed to the ion microscope of a special design, which was used to image a surface region of less than 0.5 mm^2^ [86].

SIMS has been at the forefront of the development of MSI techniques for decades. Levi-Setti et al. published an article on the progress of high-resolution scanning ion microscopy and SIMS imaging microanalysis in 1985. Kingham et al. published their work on three-dimensional SIMS imaging and the depth profiling of biological material, such as bone tissue, in 1987, showing that the ToF mass analyzer was best for imaging when compared with a quadrupole or magnetic sector due to its greater sensitivity. However, the erosion time was several orders of magnitude less, thus limiting this method to thin films and small area depth profiles [87]. One of the earliest publications on plant–microbe interactions was from Lorquin et al., where they used HPLC and GC-MS to determine nodule factors from soil bacteria interacting with host plants [88]. Since then, ToF-SIMS and another method known as nanoSIMS (see below) have been used for the imaging of plants, bacteria, and plant–microbe interactions, to analyze carbon and nitrogen assimilation by soil microbes [89], map nutrient uptake in situ in the rhizosphere [90], and image the in situ flow of photo-assimilated carbon through arbuscular mycorrhiza into root and hyphae-associated soil microbial communities [44]. 

**Figure 4:**
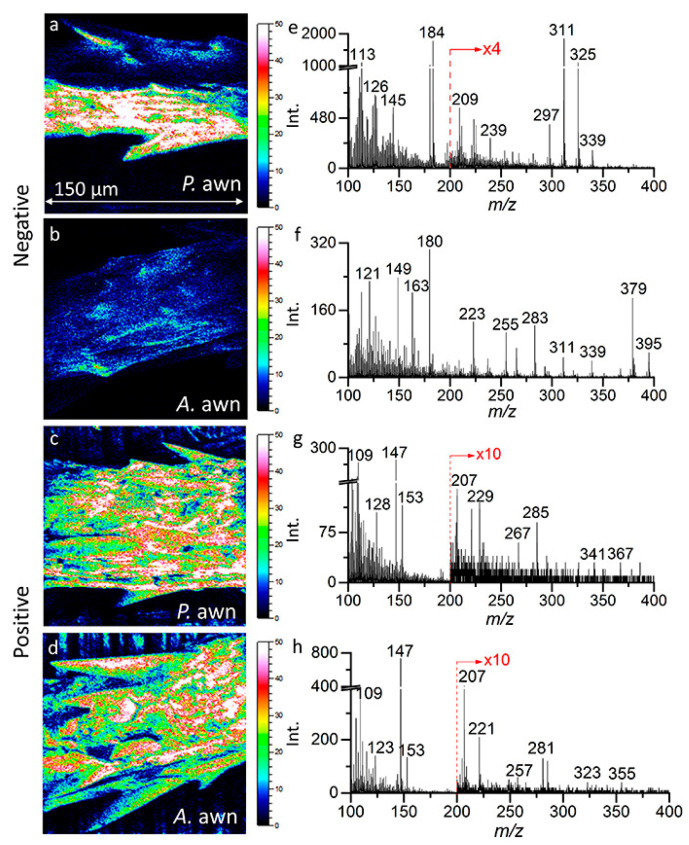
The negative ion images collected, represented by (**a**,**b**), with corresponding spectra (**e**,**f**). (**a**,**b**) are *Brachypodium* awns treated with *Pseudomonas* and *Arthrobacter*, respectively. Similarly, (**c**,**d**), with corresponding spectra (**g**,**h**), show the positive ion images, where (**c**,**d**) are *Brachypodium* awns treated with *Pseudomonas* and *Arthrobacter*, respectively. Reprinted with permission from Zhang, Y.; Komorek, R.; Zhu, Z.; Huang, Q.; Chen, W.; Jansson, J.; Jansson, C.; Yu, X.Y. Mass spectral imaging showing the plant growth-promoting rhizobacteria’s effect on the Brachypodium awn. *Biointerphases* **2022**, *17*, 031006, doi:10.1116/6.0001949 [68]. Copyright 2022, American Vacuum Society.

More recently, Zhang et al. showed that ToF-SIMS imaging could be used to study the effects of plant-growth-promoting rhizobacteria on *Brachypodium* awn. Plant-growth-promoting rhizobacteria reside within the rhizosphere [68]. *Brachypodium distachyon*, a C3 representative plant model, has been used to show plant–microbe interactions, and it is considered an ideal species for this type of experiment [25,68]. Zhang et al. studied the interaction of *Arthrobacter chlorophenolicus* and *Pseudomonas fluorescens*, planktonic cells and biofilms on the *Brachypodium* seed, which are referred to as awns. They were able to characterize plant metabolites, such as flavonoids, phenolic acids, fatty acids, and indole-3-acetic acid, on the awn surface. They also used principal component analysis (PCA) for the evaluation of the characterized metabolites and their interactions with the awn surface. The experiment determined that certain fragments of flavonoids, such as kaempferol (*m*/*z*^−^ 285, C_15_H_9_O_6_^−^) and quercetin (*m*/*z*^−^ 301, C_15_H_9_O_7_^−^), were present in only the *Pseudomonas*-treated samples and suggested that flavonoids respond more against pathogenic Gram-negative species [68]. They also showed that fatty acids’ observed intensity increased when plant-growth-promoting bacteria were introduced to the seedling or awn. The PCA results determined that both Gram-negative and Gram-positive bacteria affected the awns. Figure 4 demonstrates the ability of SIMS imaging to capture the molecular and morphological information from a small uneven surface to better understand how plant-growth-promoting bacteria interact at the surface of the spermosphere. ToF-SIMS imaging here also provides elemental information on the plant–microbe interface with high sensitivity.

### 4.2. Matrix-Assisted Laser Desorption/Ionization Mass Spectrometry

MALDI mass spectrometry is a laser desorption ionization technique that involves treating the sample with a matrix substance, either solid or liquid, to enhance the ionization of the sample. The matrix is typically compounds such as dihydroxybenzoic acid (DHB) [91], sinapinic acid (SA) [92], or α-cyano-4-hydroxycinnamic acid (CHCA) [93]. MALDI has been used in several publications involving microbial interactions and it can provide information on the molecular species of the microbial communities [94]. However, MALDI analysis is highly dependent on the selection of the correct matrix for the sample, how well the laser interacts with the matrix, and the details of the sample preparation prior to analysis. It is critical when selecting the matrix that one understands the absorption range so that it is compatible with the laser. Incompatible matrix treatment will provide little to no useful ion signal from the sample. The absence of ion signal can result from insufficient desorption and/or ionization of the analyte, and one often does not know the reason for the failure. Additionally, it is crucial when a matrix is applied that there is co-crystallization with the sample, so that the laser pulses will desorb and ionize not only the matrix but the sample molecules as well [92]. Co-crystallization provides an equal distribution of sample and matrix and allows the laser to easily desorb the material from the sample surface, thus increasing the ionization and ion yield. CHCA is typically used for small molecules and peptide samples, whereas DHB is used for lipids. The latter can be used for small molecules and SA is primarily used for protein analysis [17].

MALDI is most commonly coupled to a ToF mass analyzer, which works by extracting the ions generated under a vacuum in the sample chamber and introducing them into the flight tube. Here, ions can be steered and directed with ion optics such as Einzel lenses while varying the applied voltages. As the ions travel through the flight tube, they will begin to separate based on their mass and charge ratios (*m*/*z*), leading smaller, lighter *m*/*z* ions to reach the detector first and the larger, heavier ions to follow. Orbitrap mass analyzers are also used in MALDI [52,53,54,95,96]. Orbitraps operate by trapping an ion radially around a centralized spindle electrode. As the trapped ions rotate around the spindle electrode, independent of the energy and spatial spread, the harmonic oscillations displayed as electromagnetic radiation are measured and converted into *m*/*z* values by Fourier transforms [97]. Orbitrap mass analyzers have high resolving power—for example ~160,000 at *m*/*z* 750—and mass accuracy, which enhances the chemical information obtained from the mass spectra [53].

MALDI MSI has been used for the quantification and spatial localization of organic acids in root exudates. Low-molecular-weight organic acids from root exudates were examined for their roles in plant nutrition and pH modification in the rhizosphere, and as bio-stimulants and chemoattractants, by Gomez Zepeda et al. [51]. Organic acid exudation from the plant roots to the rhizosphere was previously determined as a mechanism by which plants cope with cation toxicity. Creating methods for the rapid and precise quantification and localization of the organic acids in the plant roots has improved the understanding of their role in response to phosphate deficiency and toxic cations [51]. 

**Figure 5:**
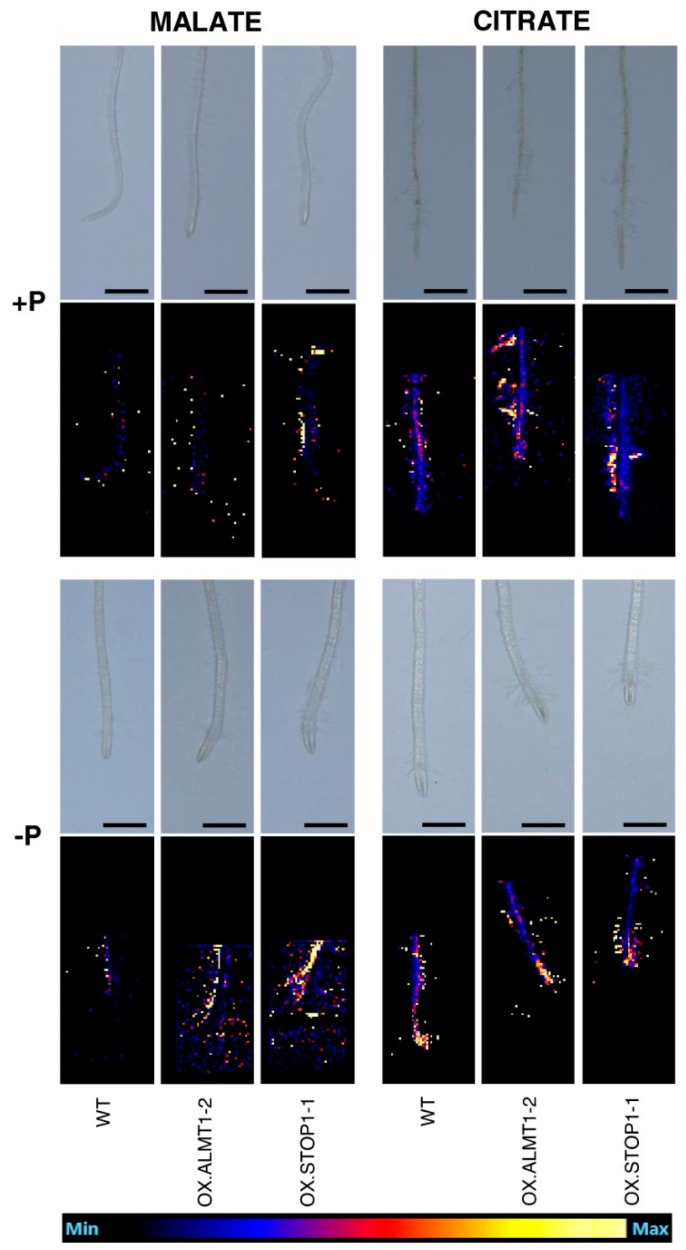
MALDI-MSI images showing the spatial distribution of malate and citrate in *Arabidopsis thaliana* roots under phosphorus abundance or reduction in wild-type and selected transgenic lines. The scale bar shown is 1 mm. Reprinted with consent from John Wiley & Sons, Inc [51].

As shown in Figure 5, MALDI was used to determine the spatial distribution and localization of malate and citrate within the plant roots of *Arabidopsis* wild-type seedlings, aluminum activated malate transporter 1 (OX.ALMT1), and positive transcriptional regulator sensitive to proton rhizotoxicity 1 (OX.STOP1), which are the transgenic lines that the *Arabidopsis* genome expresses. It was determined that the wild-type malate signal had no distribution differences in phosphate abundance or restriction. OX.ALMT1 and OX.STOP1 tended to have a wider spread in the rhizosphere and higher intensity with both phosphate abundance and restriction compared to the wild type, corroborating the findings that the overexpression of ALMT1 and STOP1 enhances malate exudation from the roots [51]. This work demonstrates that MALDI can be used to analyze and understand nutritional stresses, metal toxicity, responses to pH, and plant–microbe interactions [51].

### 4.3. Laser Desorption/Ionization Mass Spectrometry and Related Methods

LDI mass spectrometry has been around since the early 1960s, shortly after the invention of the laser in the 1950s. Levine, J. F. Ready, E. Bernal, W. I. Linlor, R. E. Honig, and J. R. Woolston were some of the researchers who first explored desorption plume dynamics and used this technique to develop high-sensitivity mass spectrometers [98,99,100,101,102,103]. As time passed and the adaptations and improvements to LDI developed, more researchers began to use it and, incidentally, it became the foundation of MALDI after early experiments from Hillenkamp et al. [104]. Since then, laser desorption has developed into an enormous field, with areas of interest in microbial and biofilm analysis [49,50,94,105,106], elemental and molecular analysis for biomaterials [33,47,57,61,107], cellular analysis [59,108], drug analysis [9,109], geological analysis [4,110], and elemental analysis and instrumentation design [48,111]. LDI is achieved by exceeding a substance’s ablation/ionization threshold, albeit by a mechanism still subject to debate: thermal vaporization, nonthermal melting, electron–lattice heating, shockwave propagation, plasma expansion, and proton or cation transfer have all been discussed as LDI mechanisms [112]. 

Laser desorption has prospered since its inception and many different methods and adaptations have become available. A key development is laser desorption postionization (LDPI). It employs two nanosecond (ns) or femtosecond (fs) pulsed laser systems for separate desorption and photoionization steps. For example, fs laser desorption postionization mass spectrometry (fs-LDPI-MS) utilizes laser pulses <100 fs for the “cold” ablation or thermal vaporization of a sample, meaning that as the energy excites the atom or molecule, the electrons will release before the energy relaxation event, allowing this to be a universal ablation method causing minimal sample damage. The desorption laser releases primary ions and neutrals into a plume and a second vacuum ultraviolet (VUV) ns laser is used to ionize the neutrals within the plume, creating positions, while the primary ions are suppressed via a biased voltage grid [4,5]. The postions are then introduced to the mass analyzer for analysis. LDPI has been used for microbial and biofilm analyses [49,50] as well as geological analyses [4]. 

Hieta et al. 2021 used laser ablation atmospheric pressure photoionization mass spectrometry (LAAPPI-MS) imaging with a 70 µm lateral resolution to analyze *Arabidopsis thaliana*’s leaf trichome and vein structures for metabolite identification and mapping [59]. LAAPPI-MS utilizes an ns pulsed mid-IR laser, the same as used in LAESI, to ablate the leaf surface to generate a plume that is intercepted by the nebulized solvent spray and photoionized by a VUV lamp, both directed towards the mass analyzer inlet. A portable version of LAAPPI-MS was demonstrated using a handheld laser and an ion trap mass spectrometer [113]. 

**Figure 6:**
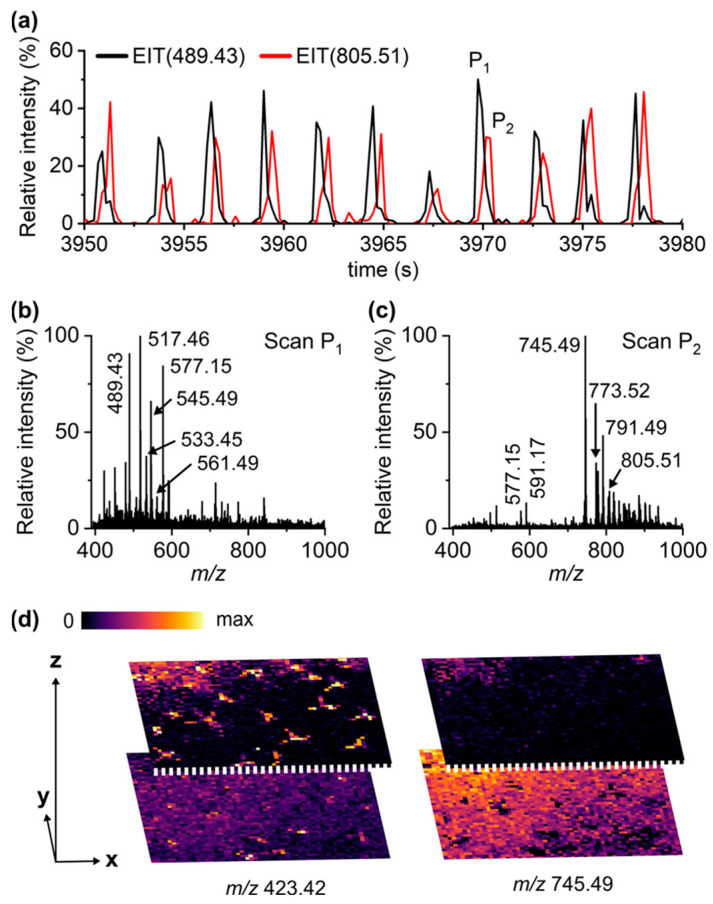
The extracted ion chromatogram demonstrated in (**a**). The different peak scans, P1 and P2, display clear differences in mass spectra, as shown by (**b**,**c**). (**d**) shows the images collected at different depths. Reprinted from Hieta, J.P.; Sipari, N.; Raikkonen, H.; Keinanen, M.; Kostiainen, R. Mass Spectrometry Imaging of Arabidopsis thaliana Leaves at the Single-Cell Level by Infrared Laser Ablation Atmospheric Pressure Photoionization (LAAPPI). *J Am Soc Mass Spectrom* **2021**, *32*, 2895–2903, doi:10.1021/jasms.1c00295 [59]. Creative Commons attribution, no alterations made.

Hieta et al. expanded the LAAPPI-MS strategy to image single trichome cells and map the trichome base, leading to the mapping of different metabolite compounds, such as kaempferol, quercetin, isorhamnetin, rhamnose, and glucose [59]. They acquired mass spectra for many lipids within the leaf lamina region and they differed from the trichome region. Most noticeably, they were able to show that LAAPPI-MS can perform the accurate depth profiling of plant material and accurately spatially resolve different structures at different depths within the leaf. Not only do the spectra results show that there is a difference between depth profile peak scans P1 and P2, but the images show that trichomes can be clearly seen at different depths at *m*/*z* 423.42, which further promotes this as a suitable imaging technique (see Figure 6).

### 4.4. Electrospray Ionization, Desorption Electrospray Ionization, and Laser Ablation Electrospray Ionization Mass Spectrometry

Electrospray ionization (ESI) is the most widely used ionization method for non-volatile organic and biomolecules that are introduced by a liquid feed by direct injection or a liquid chromatograph [22]. The liquid sample is introduced into a hypodermic needle held at a high voltage under atmospheric pressure, and the resulting field at the tip of the needle disperses the sample as a charged spray driven by Columbic forces [114]. The charged spray passes into a mass spectrometer interface containing ion optical components and a series of stages of reduced pressure ending in the mass analyzer. A counter current bath gas flowing around the hypodermic needle is used to expedite the charged droplet’s evaporation, causing the droplet to become smaller, leading to an increased surface charge, as the droplets and ions then pass into a vacuum. Once the critical Rayleigh limit is reached, a Coulomb explosion event takes place, shredding the droplet and producing smaller droplets. They are sometimes referred to as daughter droplets, which then evaporate and create quasi-ions that can be analyzed [50,114]. ESI is considered a soft ionization technique since it leads to less sample fragmentation than electron impact ionization (used on gas feeds from a gas chromatograph). 

Desorption electrospray ionization (DESI) impinges a steam of highly charged solvent droplets from an ESI source onto a solid sample: the scattered species containing the analyte are then captured by the mass spectrometry interface [32,58]. Samples analyzed by DESI are held at atmospheric pressure, whereas MALDI, SIMS, and LDI/LDPI are operated typically under a vacuum [13]. DESI has become increasingly used for the analysis of the plant rhizosphere since it is not limited to sample preparation for vacuum analysis. However, DESI has a relatively poor lateral resolution when compared to the other ionization techniques [22,57]. Laser ablation electrospray ionization (LAESI) was developed by Vertes and coworkers by combining a mid-infrared pulsed laser that excites water in a sample, leading to the ablation of neutrals in a plume [115]. The ablation plume then crosses an electrospray flow, which postionizes the neutrals in a manner similar to traditional ESI. LAESI is conceptually like MALDI, except that water inherent in the sample acts as the matrix. LAESI has a higher lateral resolution than DESI, but a lower lateral resolution than MALDI. A variant is laser ablation atmospheric pressure photionization (LAAPPI), which uses a different method for the postionization of the neutrals in the ablation plume [22,57]. Plant analysis is amenable to LAESI and LAAPPI, but the signal can vary with the water content (i.e., a lower signal is expected from regions of samples with higher cellulosic content). 

**Figure 7:**
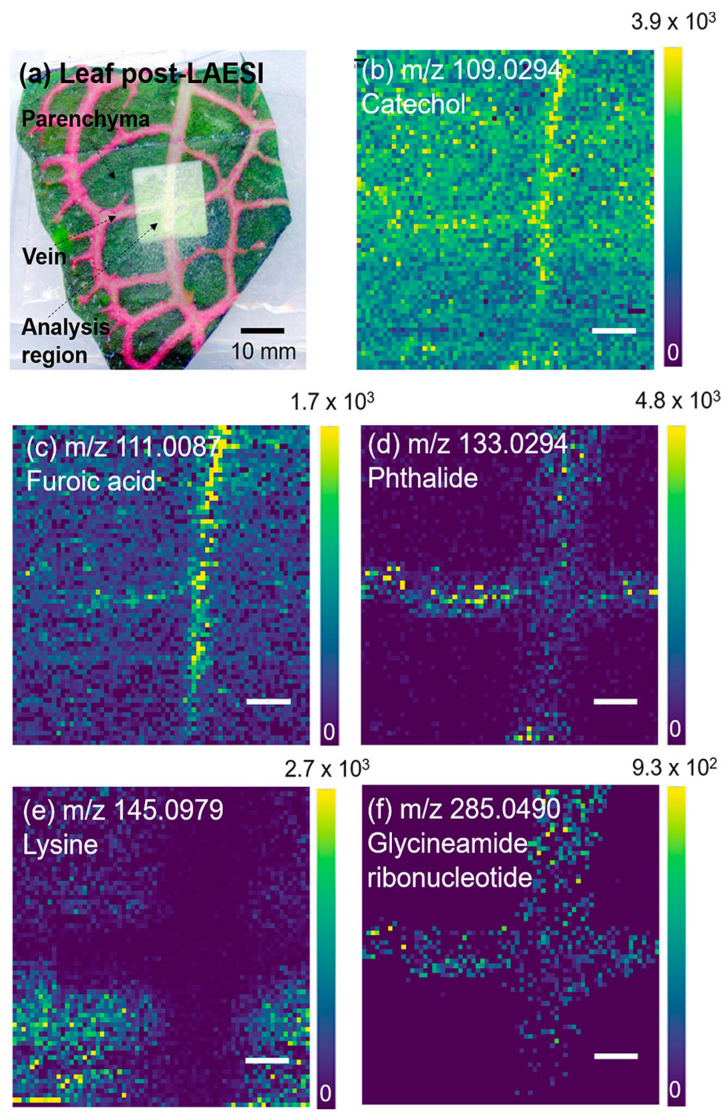
Laser ablation electrospray ionization (LAESI) MS images of *Fittonia argyroneura* leaf with spatial resolution of 40 µm. Figure 4a is an optical image post-mass spectrometry imaging, showing the lighter region to be the region of interest. Figure 4b–f show the ion images for catechol, furoic acid, phthalide, lysine, and glycinamide ribonucleotide. All ions are emitted from within the vein, except for lysine, which is from the parenchyma. Scale bar is 200 µm. Reprinted (adapted) with permission from Taylor, M.J.; Liyu, A.; Vertes, A.; Anderton, C.R. Ambient Single-Cell Analysis and Native Tissue Imaging Using Laser-Ablation Electrospray Ionization Mass Spectrometry with Increased Spatial Resolution. *J Am Soc Mass Spectrom* **2021**, *32*, 2490-2494, doi:10.1021/jasms.1c00149 [10]. Copyright 2023 American Chemical Society.

Taylor et al. used high-resolution MSI to obtain 40 µm spatial resolution images of *Fittonia argyroneura* leaves, and they were able to identify chemical species specific to the physical structure within the plant leaf [10]. The mass detector used for this experiment was an Orbitrap, which allowed for a mass resolution of ~60,000 but only slow image acquisition times. Taylor et al. were able to desorb and analyze samples from distances of 6–80 mm. The *Fittonia argyroneura* leaf was sectioned into 1.5 cm^2^ pieces, which included the veins and mesophyll, for MSI detection. The analysis produced metabolite-rich spectra and identified catechol, furoic acid, phthalide, lysine, and glycinamide ribonucleotide. As shown in Figure 7, all the metabolites identified were shown within the veins, except lysine, which was present only in the mesophyll. Lysine within the mesophyll could contribute to the lysine acetylation of proteins during photosynthesis [10]. Overall, these results show that LAESI is a versatile method for the analysis of metabolites from plant species using MSI, with the ability to analyze plant–microbe interactions.

## 5. Machine Learning for ToF-SIMS and MALDI Data Analysis

Mass spectral images can have thousands of pixels, each associated with a complete mass spectrum, leading to gigabyte-sized data files [116]. Researchers often apply multivariate analysis to MSI data, including techniques such as principal component analysis (PCA), multivariate curve resolution (MCR), maximum autocorrelation factors (MAF), and non-negative matrix factorization (NMF) [33,66,116,117]. These statistical methods, which belong to the matrix factorization approach, reduce the MSI dataset to observed trends within the data, allowing the user to gain insight into the underlying meaning and structure of the MSI data [118]. Recently, more sophisticated methods of machine learning (ML), such as t-distributed stochastic neighbor embedding (t-SNE), uniform manifold approximation, projection (UMAP), and self-organizing maps (SOMs), have been used when datasets exhibit a non-linear pattern in MSI research on biological or polymeric samples [116]. ML techniques such as SOM are becoming increasingly popular, being used instead of traditional multivariate analyses such as PCA, although PCA has been utilized more widely [119].

PCA and NMF techniques are often employed with ToF-SIMS analysis in the surface analysis and one-dimensional spectral analysis of sample layers [66,120]. Recently, Gardner et al. and Madiona et al. used the ML artificial neural network technique of SOMs to interpret complex ToF-SIMS data [116,119]. Gardner et al.’s results showed that SOMs revealed accurate information relating to polymer surface chemistries and offered an intuitive approach for the visualization of the complex relationships of individual pixels and the spectra display for MSI [116]. Madiona et al. were able to highlight the discrimination of high-weight polymers with a similar structure and composition [119]. This type of discrimination could be potentially beneficial in analyzing microbe–plant interactions, with the potential to determine the metabolite secretion composed of heavy amino acids, lipids, and fatty acid chains. 

A recent study by Zhang et al. used 14 ML statistical algorithms in combination with MALDI-TOF to differentiate among thermal degradation biological materials through its intact peptidome with bovine milk [38]. Their results showed that the top four algorithms used had values of accuracy, prediction, and recall above 0.96. This finding indicates that using ML techniques in tandem with MALDI-MS can provide fast and accurate methods to classify and identify biological products under different thermal treatment conditions [38]. ML techniques have also been used in antimicrobial resistance screening for *Campylobacter* and *Staphylococcus* bacterial strains by MALDI [121,122]. Both experiments yielded high accuracy and precision, and the methods could be used for rapid antimicrobial resistance screening.

## 6. Conclusions and Outlook

The elucidation of plant–microbe interactions can improve the understanding of their impacts on the environment and agriculture, as well as their roles in microbial sustainability and survivability mechanisms. However, such interactions are difficult to analyze or characterize. MSI has been shown to be a useful tool in deciphering some of these processes, to map nutrient flows and metabolite distributions. The different regions of a plant contain diverse varieties of microbes, each with distinguishing effects on the plant. The interpretation of the ways in which the plants interact with bacteria can be accomplished using MALDI, LAESI, SIMS, DESI, LDI, and other MSI techniques. 

MSI is a field that is constantly adapting, and new innovations are made continuously. Until the past decade, there was no suitable method to image the vacuum–liquid interface, which has increased the amount of useful information gained from these plant-microbe interactions. New laser-based systems and atmospheric pressure instruments have continued to develop, leading to more applications for the analysis of these types of samples. Tandem mass spectrometry and multimodal imaging methods are becoming more frequently used in conjunction with one another for problems central to plant–microbe interactions. Combining transmission electron microscopy (TEM) or confocal laser scanning microscopy (CLSM) with MSI has shown the benefits of multimodal imaging and continues to push the boundaries of imaging sciences. There has been a surge in recent efforts to explore existing machine learning models in the analysis of MSI datasets. Upcoming developments to machine learning and artificial intelligence models are expected to accelerate the analysis of large MSI datasets in a more efficient manner. 

MSI provides molecular information about the bacterial–host interface, metabolite mapping and identification, and depth analysis, all while having a spatial resolution of 0.2–200 µm. When compared with the more established bulk omic techniques or optical imaging techniques (e.g., CLSM), MSI offers valuable spatial chemical mapping and molecular-level information from the community to the single-cell level. Although no single technique can cover the entire spatial scale or accomplish every task, it is anticipated that MSI techniques will continue to evolve to expand the understanding of these interactions.

## Data Availability

No new data were created or analyzed in this study. Data sharing is not applicable to this article.

## Glossary

AP	Atmospheric Pressure
CHCA	Cyano Hydroxycinnamic Acid
CLSM	Confocal Laser Scanning Microscopy
DESI	Desorption Electrospray Ionization
DHB	Dihydroxybenzoic Acid
ESI	Electrospray Ionization
fs	Femtosecond
FT-ICR	Fourier Transform Ion Cyclotron Resonance
GC-MS	Gas Chromatography Mass Spectrometry
HPLC	High-Pressure Liquid Chromatography
IR	Infrared
LAAPPI	Laser Ablation Atmospheric Pressure Photoionization
LAESI	Laser Ablation Electrospray Ionization
LAPPI-MS	Laser Ablation Atmospheric Pressure Photoionization Mass Spectrometry
LDI	Laser Desorption Ionization
LDPI	Laser Desorption Postionization
*m*/*z*	Mass-to-Charge Ratio
MAF	Maximum Autocorrelation Factors
MALDI	Matrix-Assisted Laser Desorption Ionization
MCR	Multivariate Curve Resolution
ML	Machine Learning
MS	Mass Spectrometry
MSI	Mass Spectrometry Imaging
NMF	Non-Negative Matrix Factorization
ns	Nanosecond
PCA	Principal Component Analysis
QToF	Quadrupole Time-of-Flight
SA	Sinapinic Acid
SALVI	System for Analysis at the Liquid–Vacuum Interface
SIMS	Secondary Ion Mass Spectrometry
SNMS	Secondary Neutral Mass Spectrometry
SOM	Self-Organizing Map
TEM	Transmission Electron Microscopy
ToF	Time-of-Flight
ToF-SIMS	Time-of-Flight Secondary Ion Mass Spectrometry
t-SNE	t-Distributed Stochastic Neighbor Embedding
UHV	Ultra-High Vacuum
UMAP	Uniform Manifold Approximation Projection
UV	Ultraviolet
VUV	Vacuum Ultraviolet
